# Parry–Romberg Syndrome with Uhthoff's Phenomena: A Spectrum of Autoimmune Disease?

**DOI:** 10.1155/2019/1752456

**Published:** 2019-04-18

**Authors:** Samuel Asanad

**Affiliations:** ^1^David Geffen School of Medicine, University of California, Los Angeles, USA; ^2^Doheny Eye Institute, Los Angeles, California, USA

## Abstract

Parry–Romberg syndrome (PRS) is a rare disorder characterized by unilateral facial atrophy. Currently, the pathogenesis of PRS is poorly understood and no definitive treatment is available. This article reports the case of a 51-year-old woman with progressive hemifacial atrophy following herpes zoster infection, who presented with a concomitant chronic history of heat-induced diplopia. Magnetic resonance imaging showed unilateral cerebral white matter, periventricular, and medial longitudinal fasciculus lesions. The patient's diplopia resolved following treatment with valacyclovir. Infection has been previously considered as potential cause of PRS. However, herpes-induced PRS with ophthalmologic manifestations of Uhthoff's phenomena has not previously been reported. The present case suggests that PRS may possibly have an autoimmune etiology resembling that of multiple sclerosis.

## 1. Introduction

Parry–Romberg syndrome (PRS), or progressive hemifacial atrophy, is a rare and poorly understood condition characterized by unilateral facial atrophy commonly affecting the skin, subcutaneous tissue, and muscles; and occasionally extending to osteocartilaginous structures [1–5]. PRS most commonly presents in the first to second decades of life and has an incidence of 1 in 700 with a female preponderance. Ocular manifestations, although less common in PRS, can develop either before, during, or long after the onset of facial atrophy [6–12]. The pathophysiology of PRS is unknown; however autoimmune, infectious, traumatic, endocrinologic, and genetic etiologies have been postulated [13].

As the most common ocular manifestation of PRS, enophthalmos develops from retro-orbital fat or bone atrophy [14]. Patients with PRS may present with diplopia secondary to restricted ocular motility resulting from either ocular motor nerve dysfunction, extraocular muscle degeneration, or fibrosis [15]. This article presents the case of a female patient with herpes-induced PRS, who presented with heat-induced diplopia suggestive of Uhthoff's phenomena and an internuclear ophthalmoplegia (INO) involving the medial longitudinal fasciculus (MLF). These findings imply that PRS may have a disease course similar to multiple sclerosis (MS) and that PRS may possibly have an autoimmune etiology resembling that of MS.

## 2. Case Presentation

A 51-year-old woman with PRS presented with an 8-year history of binocular horizontal diplopia on right lateral gaze exacerbated during exercise. Her diplopia on exertion would resolve shortly after cessation of exercise while cooling off. The patient denied eye pain, floaters, photopsia, decreased vision, paresthesias, or motor deficits of the extremities. The patient's history of PRS was characterized as progressive left-sided facial atrophy for a period of 10 years following infection with herpes zoster and postherpetic neuralgia in the distribution of the ophthalmic division (V1) of the trigeminal nerve. Examination showed atrophy involving the left temporal region and alopecia localized to the left frontal parietal region, corresponding with V1. There was minimal hyperpigmentation of skin over the left vivum dermatome and a linear hypopigmented scar (*coup de sabre*) was observed. On neurologic examination, speech was fluent without dysarthria or aphasia, and cognitive functions were intact. Tongue and uvula were midline. Motor examination showed normal tone, no evidence of drift, and 5/5 strength bilaterally. Coordination and gait were intact. Deep tendon reflexes were 2+ bilaterally and plantar responses were flexor. Sensory examination was normal and intact to light touch and pin prick testing. The patient's past medical history was significant for Hashimoto's thyroiditis, migraine headaches, and recurrent outbreaks of herpes simplex labialis. Medications included levothyroxine 50 mcg daily. Family history was significant solely for migraines in the patient's sister.

On ophthalmologic examination, best-corrected visual acuity was 20/20 in both eyes. Applanation tonometry measured intraocular pressures of 12 in both the right and the left eyes. Fundus examination was normal appearing without evidence of pallor or edema. Pupils were equal and reactive to light with no afferent pupillary defects. Color vision assessment on Ishihara plate testing showed 15/15 OD and 13/15 OS. Ocular motility examination was significant for 30-degree limited adduction of the left eye on right lateral gaze.

**Figure 1:**
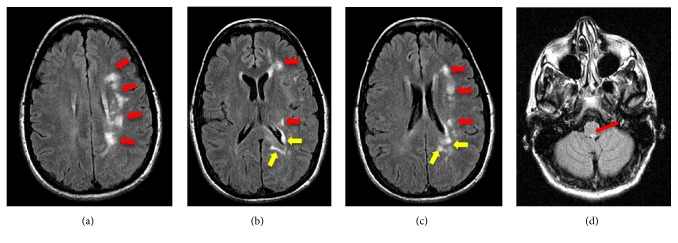
Axial brain MRI (a-d) depicts left-sided (a-c) cerebral white matter lesions (red arrows), periventricular lesions (b and c) of the left frontal and posterior horns of the lateral ventricles (yellow arrows), and T2-hyperintense foci of the midbrain (d) corresponding to the medial longitudinal fasciculus (red arrow).

**Figure 2:**
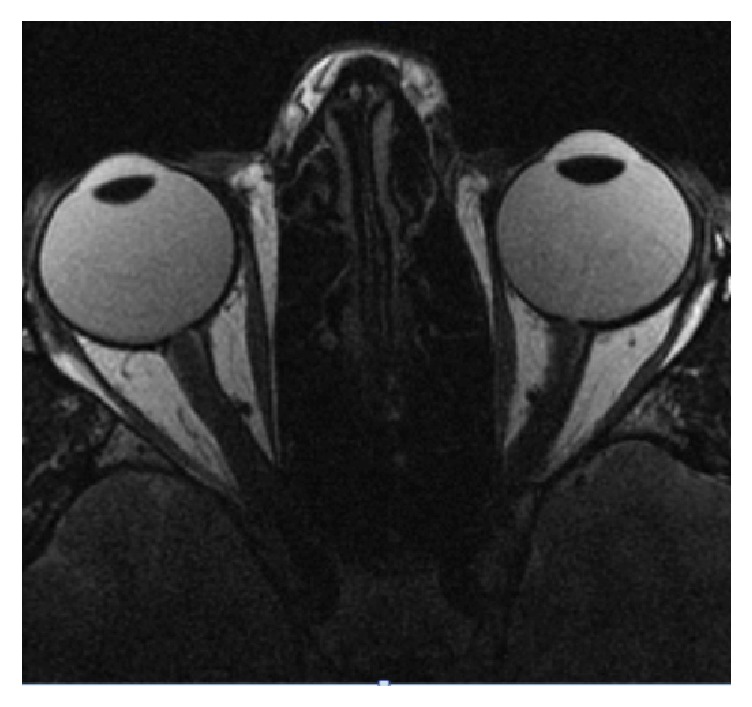
MRI scan of the orbits depicting normal extraocular muscles bilaterally without evidence of fibrosis or atrophy.

Autoimmune panel was positive for antinuclear antibody (ANA) (1:40 speckled pattern, normal range <1:40) and negative for beta 2 glycoprotein and cardiolipin immunoglobulins (IgA, IgG, and IgM). Vitamin B12 and Vitamin E levels were within normal limits. Magnetic resonance imaging (MRI) of the brain showed multiple white matter lesions including the left frontal lobe, left parieto-occipital area, and periventricular areas of the left frontal and left posterior horns of the lateral ventricles (Figures 1(a)–1(c)). In addition, FLAIR/T2 hyperintensity of the midbrain region corresponding to the area of the MLF was seen (Figure 1(d)). MRI of the orbits (Figure 2) showed normal positioning of the globes bilaterally without evidence of proptosis or extraocular muscle fibrosis or atrophy. MRI of the spinal cord showed no evidence of spinal lesions.

Given recurrent outbreaks of herpes simplex labialis, the patient was started on valacyclovir 500 mg PO BID. On follow-up 3 months later, the patient indicated that her heat-induced diplopia was completely resolved. Ocular motility examination was significantly improved with less than 10-degree limited adduction of the left eye on right lateral gaze.

## 3. Discussion

PRS pathogenesis is heavily debated and our understanding of the periocular, ocular, and systemic presentations of PRS remains unclear. This report presents the case of a female patient, who acquired PRS following herpes zoster infection and later presented with heat-induced diplopia secondary to INO involving the MLF. Currently, only three cases of INO associated with herpes zoster have been reported. For the first time, the present article reports a case of INO associated with herpes zoster in a patient with PRS.

An autoimmune etiology for PRS has been previously suggested. Studies have shown inflammatory changes on brain biopsy neuropathology, co-occurrence of autoimmune disorders, positive serum autoantibodies, and symptomatic resolution with immunosuppressive therapies [13]. Infectious causes of PRS, including herpes, have also been suggested by previous reports [13]. The current patient had a co-occurring autoimmune disorder of Hashimoto's thyroiditis and a positive ANA autoantibody titer. In addition, the patient's PRS followed a herpetic infection in the ophthalmic division of the trigeminal nerve with subsequent facial atrophy in the corresponding region. MRI revealed cerebral and periventricular white matter lesions, a hallmark pattern of MS. Intriguingly, the current patient presented with heat-induced horizontal diplopia on right lateral gaze with radiographic evidence of INO involving the MLF, a clinical presentation also consistent with MS. A recent report by Zhang et al. showed that PRS may have a disease course similar to the “relapsing-remitting” subtype of MS as triggered by a chronic hepatitis B virus infection [14]. Infiltration of the central nervous system by immune cells and microbial infections likely serve as immunological triggers for demyelination in MS [15]. Infectious causes of INO commonly include tuberculosis, AIDS, brucellosis, cysticercosis, and syphilis [16]. In a report by Caroll et al., herpes-induced INO was likely associated with a demyelinating process involving the brainstem [17]. Taken together, it is plausible that PRS may have an autoimmune etiology resembling that of MS.

Consistent with Zhang et al., the present case further supports the notion that PRS and MS may possibly fall along a continuous spectrum of autoimmune disease [14]. In the current case, the white matter lesions localized to the left hemisphere, the same side as the patient's facial atrophy. These unilateral facial and intracranial manifestations of disease are consistent with the radiographic findings similarly reported by Cory et al. [18]. Notably, the current patient's ophthalmologic symptoms of heat-induced diplopia resolved following valacyclovir treatment. These findings suggest that PRS may have an autoinflammatory disease course, which was possibly triggered following infection with herpes zoster. The patient's clinical findings and ocular symptoms resolved following treatment of her underlying infection. Further intervention including lumbar puncture testing was not indicated or pursued since the patient remained asymptomatic. Nevertheless, cerebrospinal fluid analysis in patients with PRS presenting with Uhthoff's phenomena may be warranted in future studies. Given that no effective treatments are currently available, an understanding of PRS pathophysiology in the context of an autoimmune etiology may provide valuable insight for deriving potential future therapies.
